# Expression of tung tree diacylglycerol acyltransferase 1 in *E. coli*

**DOI:** 10.1186/1472-6750-11-73

**Published:** 2011-07-11

**Authors:** Heping Cao, Dorselyn C Chapital, Jay M Shockey, K Thomas Klasson

**Affiliations:** 1 Commodity Utilization Research Unit, Southern Regional Research Center, Agricultural Research Service, U.S. Department of Agriculture, 1100 Robert E. Lee Blvd., New Orleans, Louisiana 70124, USA

## Abstract

**Background:**

Diacylglycerol acyltransferases (DGATs) catalyze the final and rate-limiting step of triacylglycerol (TAG) biosynthesis in eukaryotic organisms. Database search has identified at least 59 DGAT1 sequences from 48 organisms, but the expression of any DGAT1 as a full-length protein in *E. coli *had not been reported because DGAT1s are integral membrane proteins and difficult to express and purify. The objective of this study was to establish a procedure for expressing full-length DGAT1 in *E. coli*.

**Results:**

An expression plasmid containing the open reading frame for tung tree (*Vernicia fordii*) DGAT1 fused to maltose binding protein and poly-histidine affinity tags was constructed and expressed in *E. coli *BL21(DE3). Immunoblotting showed that the recombinant DGAT1 (rDGAT1) was expressed, but mostly targeted to the membranes and insoluble fractions. Extensive degradation also occurred. Nonetheless, the fusion protein was partially purified from the soluble fraction by Ni-NTA and amylose resin affinity chromatography. Multiple proteins co-purified with DGAT1 fusion protein. These fractions appeared yellow in color and contained fatty acids. The rDGAT1 was solubilized from the insoluble fraction by seven detergents and urea, with SDS and Triton X-100 being the most effective detergents. The solubilized rDGAT1 was partially purified by Ni-NTA affinity chromatography. PreScission protease digestion confirmed the identity of rDGAT1 and showed extensive precipitation following Ni-NTA affinity purification.

**Conclusions:**

This study reports the first procedure for expressing full-length DGAT1 from any species using a bacterial expression system. The results suggest that recombinant DGAT1 is degraded extensively from the carboxyl terminus and associated with other proteins, lipids, and membranes.

## Background

The functions of a considerable percentage of the proteins encoded by the sequenced genomes of many organisms including human, mouse, *Arabidopsis*, and rice are not clear. The immediate task of post-genomic biology is to determine the biological functions of proteins coded for by these genes. Many endogenous proteins occur in extremely low abundance but recombinant protein can be used as alternative sources to endogenous proteins for studying protein structure and function [1], for making high-titer antibodies [2-4], and for producing pharmaceutical reagents. However, a great number of proteins are difficult to express due to protein insolubility, protein degradation, and low-level protein expression [5]. Therefore, production of high quality recombinant protein requires optimization of protein expression and purification procedures.

Diacylglycerol acyltransferases (DGATs) are responsible for the last and rate-limiting step of triacylglycerol (TAG) biosynthesis in eukaryotic organisms. DGAT genes have been isolated from many organisms [6]. At least two forms of DGATs are present in mammals [7,8] and plants [9,10] with additional forms reported in burning bush (*Euonymus alatus*) [11] and peanut [12]. DGAT isoforms have nonredundant functions in TAG biosynthesis in plants such as tung tree (*Vernicia fordii*) [10] and animals such as mice [13]. Tung tree genome has two well-known DGAT isoforms (DGAT1 and DGAT2) which are targeted to distinct subdomains of the ER membrane, and likely carry out different roles in seed lipid metabolism [10]. Mice deficient in DGAT1 are viable, have modest decreases in TAG, and are resistant to diet-induced obesity [14,15]. Mice deficient in DGAT2 have severe reduction of TAG and die shortly after birth [13]. The fact that DGAT1 is unable to compensate for the deficiency in DGAT2 knockout mice indicates that each DGAT isoform has unique functions in TAG biosynthesis during mammal development.

**Figure 1:**
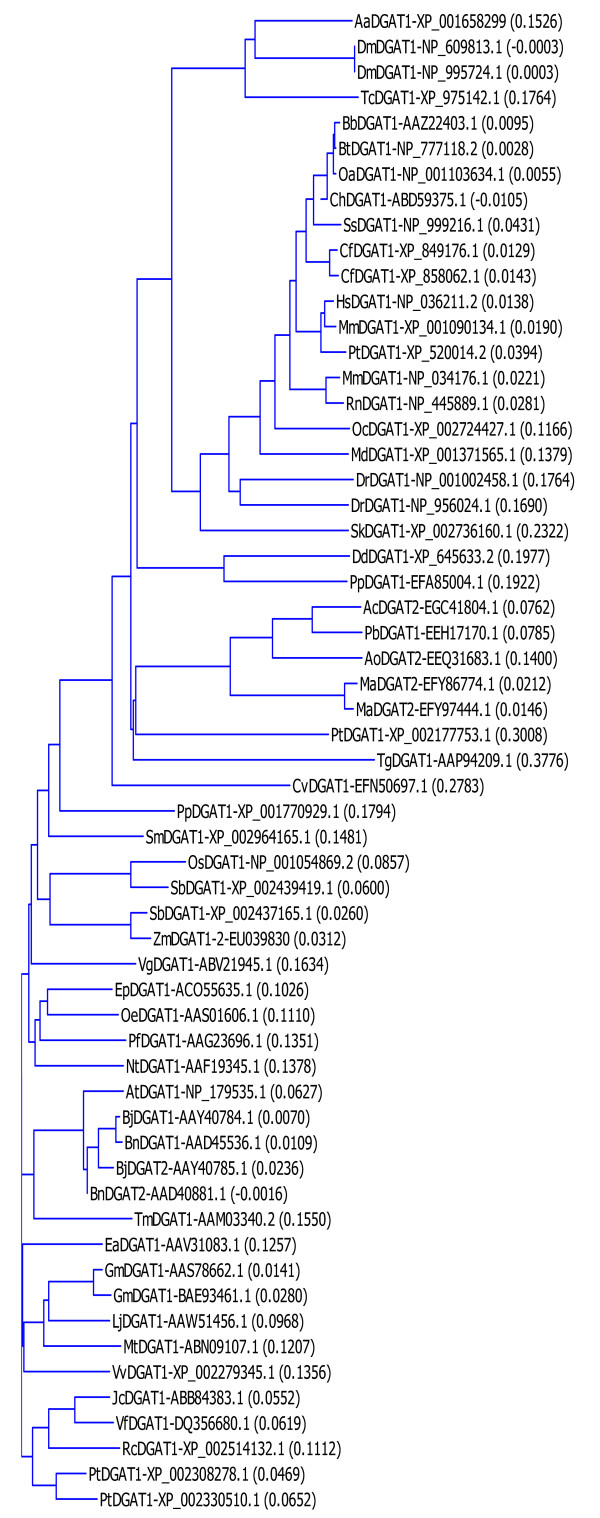
**Phylogenetic analysis of DGAT1s**. The evolutionary relationships among the 59 DGAT1s from 48 organisms were analyzed by phylogenetic analysis based on the Neighbor-Joining method of Saitou and Nei [35]. The name of each protein sequence consists of the initials of the organism followed by the assigned subfamily of DGATs in the databases and the GenBank accession number. The numbers in the parenthesis following DGAT names are the calculated distance values, which reflect the degree of divergence between all pairs of DGAT sequences analyzed. The abbreviations of the organisms are: Ac, *Ajellomyces capsulatus*; Ao, *Arthroderma otae*; At, *Arabidopsis thaliana*; Bb, *Bubalus bubalis*; Bj, *Brassica juncea*; Bn, *Brassica napus*; Bt, *Bos taurus*; Cf, *Canis familiaris*; Cv, *Chlorella variabilis*; Dd, *Dictyostelium discoideum*; Dm, *Drosophila melanogaster*; Dr, *Danio rerio*; Ea, *Euonymus alatus*; Ep, *Echium pitardii*; Gm, *Glycine max*; Hs, *Homo sapiens*; Jc, *Jatropha curcas*; Lj, *Lotus japonicas*; Ma in MaDGAT1-EFY86774.1, *Metarhizium acridum*; Ma in MaDGAT1-EFY97444.1, *Metarhizium anisopliae*; Md, *Monodelphis domestica*; Mm in MmDGAT1-XP_001090134.1, *Macaca mulatta*; Mm in MmDGAT1-NP_034176.1, *Mus musculus*; Mt, *Medicago truncatula*; Nt, *Nicotiana tabacum*; Oa, *Ovis aries*; Oc, *Oryctolagus cuniculus*; Oe, *Olea europaea*; Os, *Oryza sativa*; Pb, *Paracoccidioides brasiliensis*; Pf, *Perilla frutescens*; Pp in PpDGAT1-EFA85004.1, *Polysphondylium pallidum*; Pp in PpDGAT1-XP_001770929.1, *Physcomitrella patens*; Pt in PtDGAT1-XP_520014.2, *Pan troglodytes*; Pt in PtDGAT1a-XP_002308278.1, *Populus trichocarpa*; Rc, *Ricinus communis*; Rn, *Rattus norvegicus*; Sb, *Sorghum bicolor*; Sk, *Saccoglossus kowalevskii*; Sm, *Selaginella moellendorffii*; Ss, *Sus scrofa*; Tc, *Tribolium castaneum*; Tg, *Toxoplasma gondii*; Tm, *Tropaeolum majus*; Vf, *Vernicia fordii*; Vg, *Vernonia galamensis*; Vv, *Vitis vinifera*; Zm, *Zea mays*.

Database search has identified at least 59 DGAT1 sequences from 48 organisms (Figure 1). However, there is a gap of knowledge between the gene sequences and the proteins coded for by the DGAT genes [6]. Although DGAT expression was reported in several studies, only limited success has been achieved due to the facts that DGATs are integral membrane proteins [10,16] and are difficult to express and purify using heterologous hosts [17,18]. Information regarding the expression of DGAT1 gene in *E. coli *is limited. The amino terminal 116 amino acid residues of oilseed rape (*Brassica napus*) DGAT1 was expressed as a His-tag protein in *E. coli *[18]. Similar studies reported on the production of the amino terminal 95 residues of mouse DGAT1 in *E. coli *[19]. However, the expression of DGAT1 as a full-length protein in *E. coli *had not been reported. The objective of this study was to develop a reliable procedure for the expression of a full-length DGAT1 in *E. coli *using tung tree DGAT1 (one of the two isoforms from tung tree) as the model protein.

## Results

### Construction of bacterial expression plasmid

**Figure 2:**
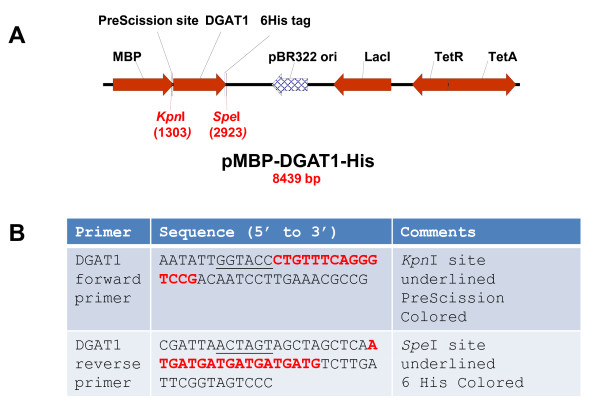
**Plasmid map used for the construction of an *E. coli *expression vector and primer sequences used for PCR-amplification of the DGAT1 insert**. (A) Diagram of the expression plasmid pMBP-DGAT1-His. Plasmid pMBP-hTTP [1] was used to express full-length DGAT1 in *E. coli*. Tung DGAT1 DNA was subcloned as described in the Materials and Methods section. (B) Primers for construction of the *E. coli *expression plasmid. The sequences for restriction enzyme digestion sites are underlined. DGAT1 forward primer and reverse primer have sequences coding for a PreScission protease digestion site and 6 histidine residues, respectively.

Plasmid pMBP-DGAT1-His was engineered to express the full-length *Vernicia fordii *(tung tree) DGAT1 in an *E. coli *protein expression system. The recombinant protein MBP-DGAT1-His (rDGAT1) contained a MBP (maltose binding protein) at the amino terminus and 6 histidine residues (His) at the carboxyl terminus (Figure 2). A PreScission protease cleavage site was engineered between the MBP and DGAT1 fusion partners. The "Methods" section describes the details of the plasmid construction.

### Degradation of recombinant DGAT1 in the soluble fraction of *E. coli*

The expression of rDGAT1 was induced by IPTG in *E. coli *strain BL21(DE3) and detected by immunoblotting using anti-MBP-hTTP and anti-MBP-mTTP polyclonal antibodies, which were raised in rabbits against purified recombinant human and mouse TTP proteins fused to MBP [2,3]. These antibodies have been shown to react with MBP and MBP fusion proteins with high specificity [2,3].

**Figure 3:**
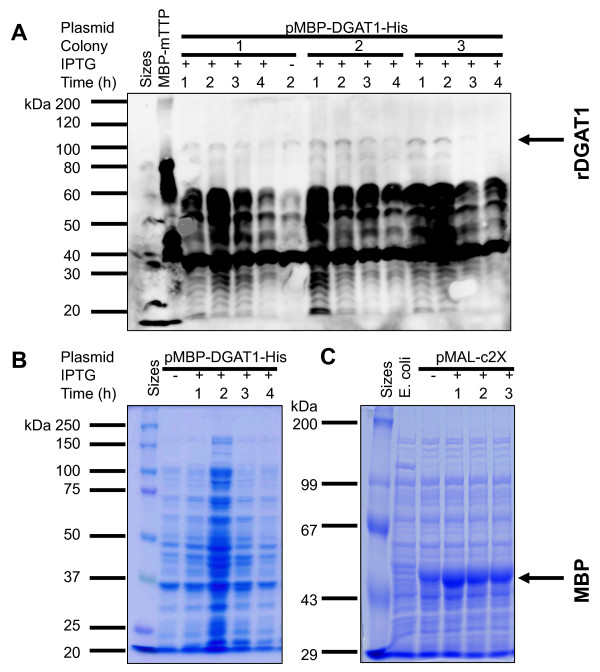
**Degradation of recombinant DGAT1 in the soluble fraction of *E. coli***. Plasmids pMBP-DGAT1-His and the empty vector pMAL-c2X were transformed into *E. coli *BL21(DE3). Protein expression was induced by IPTG for various times. "+" and "-" represent protein induction with or without IPTG. SDS-PAGE (10%) was used for the separation of proteins. (A) Immunoblotting detection of rDGAT1. Recombinant DGAT1 in the 10,000*g *supernatant of *E. coli *transformed with pMBP-DGAT1-His was identified by polyclonal antibodies raised against MBP-mTTP fusion protein [2,3]. Partially purified MBP-mTTP was used as a positive control for immunoblotting. The full-length rDGAT1 is marked with an arrow. (B) Coomassie brilliant blue staining of proteins in the 10,000*g *supernatants of *E. coli *transformed with pMBP-DGAT1-His. (C) Coomassie brilliant blue staining of proteins in the 10,000*g *supernatants of *E. coli *transformed with pMAL-c2X as an empty vector control for the expression of MBP.

Anti-MBP-mTTP antibodies detected a number of bands ranging from approximately 40 kDa (corresponding to that of MBP, 42 kDa) to 60 kDa and a trace amount of protein corresponding to the full-size rDGAT1 (909 amino acid residues, 102 kDa) (Figure 3A). There was no significant difference in protein expression levels among different starting colonies of the bacterium. The expression levels, judged from the overall full-length and degradation products, were decreased under longer expression time tested (Figure 3A). Less but similar sizes of the full-length and degradation products were detected in the cells without IPTG induction (Figure 3A), indicating a high basal level expression of the recombinant protein under uninduced conditions. The 60 kDa band corresponds to protein smaller than the 80-kDa-sized MBP-mTTP fusion protein (a positive control for the antibodies in lane 2 of Figure 3A). Recombinant DGAT1 expression was not significantly different when protein expression was induced at 25°C or 37°C, nor under culture medium with or without 0.2% glucose (data not shown). Similar results were obtained using anti-MBP-hTTP antibodies and the commercial anti-MBP antibodies (data not shown). In agreement with the immunoblotting results shown in Figure 3A, a protein staining gel showed that no distinct band with a molecular mass corresponding to the full-length rDGAT1 was observed in the 10,000*g *supernatant from the uninduced and IPTG-induced cells (Figure 3B).

As a control experiment, plasmid pMAL-c2X vector was transformed into the same type of *E. coli*. Like rDGAT1 expression in the uninduced cells described above, MBP protein was clearly produced in the uninduced cells (Figure 3C). In contrast to the minimal expression of rDGAT1 in *E. coli *(Figure 3A and 3B), MBP was massively induced by IPTG in the same type of cells (Figure 3C), which was specifically recognized by the commercial anti-MBP antibodies [1] and the same anti-MBP-hTTP antibodies [2] and anti-MBP-mTTP antibodies [3] used in the current study. These results suggest that rDGAT1 was expressed well but was extensively degraded in *E. coli *under the experimental conditions.

### Localization of recombinant DGAT1 in the insoluble fraction and membranes of *E. coli*

**Figure 4:**
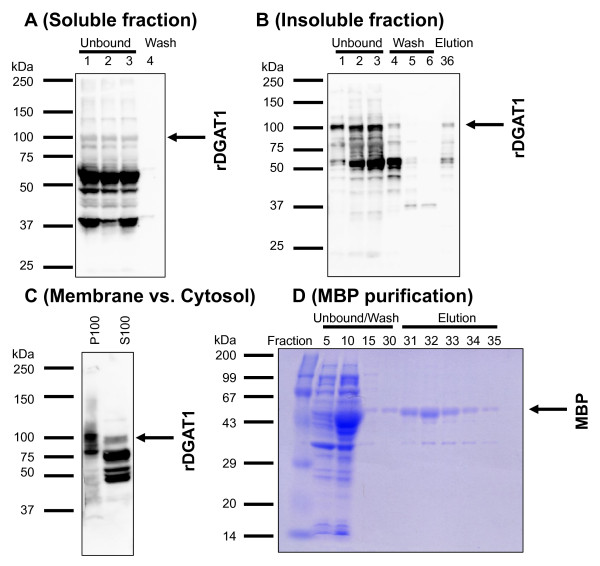
**Localization of recombinant DGAT1 in the insoluble fraction and membranes of *E. coli***. (A) Trace amount of rDGAT1 was in the soluble fraction. The 10,000*g *supernatant of *E. coli *transformed with pMBP-DGAT1-His was loaded onto an MBPTrap HP column. The column was washed extensively with amylose resin wash buffer. The bound proteins were eluted with amylose resin elution buffer containing 20 mM maltose. Proteins were separated by 10% SDS-PAGE. Recombinant DGAT1 was identified by anti-MBP-hTTP antibodies. (B) The majority of rDGAT1 was in the insoluble fraction. The 10,000*g *pellet of *E. coli *transformed with pMBP-DGAT1-His was subject to extensive sonication followed by centrifugation at 10,000*g*. This supernatant was loaded onto an MBPTrap HP column. Separation and detection of the recombinant protein was identical to those in panel A. (C) Localization of rDGAT1 in the membranes. The 10,000*g *supernatant was centrifuged at 100,000*g*. Proteins in the 100,000*g *supernatant (cytosol) and the pellet (membranes) were separated by 4-20% SDS-PAGE, transferred onto a nitrocellulose membrane, and detected by immunoblotting with anti-MBP-hTTP antibodies. S100, 100,000*g *supernatant (cytosol); P100, 100,000*g *pellet (membrane). The full-length rDGAT1 is marked with an arrow. (D) Purification of MBP from the 10,000*g *supernatant of *E. coli *transformed with pMAL-c2X as a positive control for amylose resin affinity chromatography.

Amylose resin affinity chromatography was used to identify the trace amount of the full-length protein as rDGAT1 shown in Figure 3. Based on the expression studies described above, DGAT1 fusion protein expression in *E. coli *was scaled up. The 10,000*g *supernatant was applied onto an MBPTrap HP column. After extensive washing, the bound proteins were eluted with 20 mM maltose. However, no clear protein peak was eluted with maltose as judged from UV absorbance at 280 nm (data not shown). Immunoblotting confirmed that the great majority if not all of the immuno-reactive proteins did not bind to amylose resin and was enriched in the unbound fractions (Figure 4A).

To investigate if the recombinant protein was in the insoluble fraction, the 10,000*g *pellet was sonicated extensively in the same homogenization buffer. This supernatant was used for MBPTrap column purification. The great majority of the "released" recombinant DGAT1 did not bind to the column (as in the case with the soluble rDGAT1) and was mainly recovered in the unbound fractions (Figure 4B). However, immunoblotting showed that the full-length rDGAT1 was much more enriched in the pellet than in the soluble fraction of *E. coli *and some full-length protein was eluted from the column (Figure 4A versus 4B, right lanes).

To localize the trace amount of the recombinant protein in the supernatant shown in panel A, the 10,000*g *supernatant was centrifuged at 100,000*g*. Immunoblotting showed that rDGAT1 was mainly associated with the membrane fraction and only a smaller fraction of rDGAT1 in the cytosol (Figure 4C).

Figure 4A and 4B showed that rDGAT1 bound to amylose resin poorly because little rDGAT1 was recovered in the eluted fractions. To provide a positive control for amylose resin affinity purification, *E. coli *extract with overexpressed MBP shown in Figure 3C was applied to amylose resin. Significant amounts of MBP bound to the affinity resin and was purified to near homogeneity by the affinity chromatography, although lots of the recombinant MBP did not bind to the same affinity beads (Figure 4D). These results suggest that rDGAT1 may be folded in a way to prevent MBP fusion partner from binding to the affinity resin.

### Purification of recombinant DGAT1 with Ni-NTA affinity chromatography

**Figure 5:**
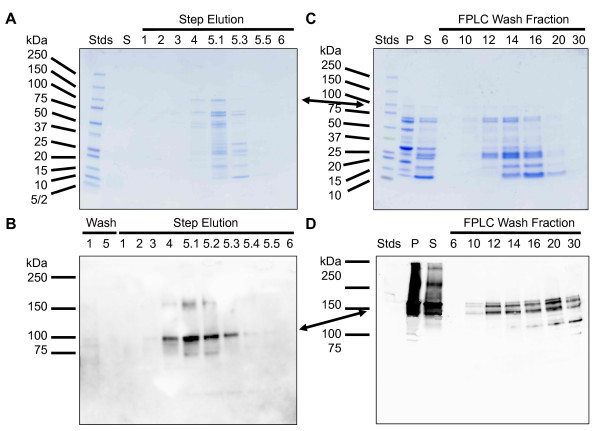
**Purification of recombinant DGAT1 from *E. coli *with Ni-NTA and amylose resin affinity chromatography**. (A, B) Ni-NTA affinity purification. The 10,000*g *supernatant was mixed with Ni-NTA affinity beads. The beads were washed five times followed by eluting the beads with imidazole solution containing 50 mM (Elution 1), 100 mM (Elution 2), 150 mM (Elution 3), 200 mM (Elution 4), 250 mM for 5 times (Elutions 5.1-5.5), and 1000 mM (Elution 6). Proteins were separated by 4-20% SDS-PAGE, stained with Coomassie brilliant blue (A) or transferred onto nitrocellulose membranes for immunoblotting with anti-MBP-hTTP antibodies (B). (C, D) Amylose resin affinity purification using Ni-NTA affinity-purified fractions. The fractions purified by Ni-NTA affinity beads as shown in panes A and B were pooled, centrifuged, and loaded onto an MBPTrap HP column. SDS-PAGE separation and immunoblotting detection of the recombinant protein was identical to those in panels A and B. S, 10,000*g *supernatant; P, 10,000*g *pellet. The full-length rDGAT1 size is marked with an arrow.

Ni-NTA beads were used to test the purification of the recombinant protein using the 10,000*g *supernatant. The bound proteins were eluted with successively increasing imidazole concentrations ranging from 50 to 1000 mM. SDS-PAGE showed that the purified fractions contained a number of proteins as shown by Coomassie blue staining (Figure 5A). The majority of the bound recombinant protein (as detected by immunoblotting) was eluted with 200-250 mM imidazole in the elution buffer and no recombinant protein was detected in the washes (Figure 5B). rDGAT1 only partially bound to Ni-NTA beads because a significant amount of the full-length rDGAT1 was recovered in the unbound fraction (data not shown). As expected, MBP and most of the degradation products were not bound to the Ni-NTA beads because the immunoreactive bands on the blot corresponded to the full-length rDGAT1 (Figures 3, 4 vs. 5B). A protein band with approximately twice the size (>150 kDa) of the full-length rDGAT1 was seen in the eluted fractions on the immunoblot after SDS-denatured gel separation (Figure 5B). A similar immunoreactive band with the higher molecular mass was also observed on other immunoblots (see below).

### Purification of recombinant DGAT1 with tandem Ni-NTA and amylose resin affinity chromatography

The proteins eluted by 250 mM imidazole from Ni-NTA affinity beads (Figure 5A) were pooled and centrifuged at 10,000*g*. The supernatant was loaded onto an amylose resin affinity column. FPLC chromatogram showed that the great majority of proteins were washed off the column and little protein was bound to the MBPTrap column (data not shown), similar to those observed using the 10,000*g *supernatant (Figure 5A). Coomassie blue staining showed that the additional affinity purification step did not improve the purity (Figure 5C). Immunoblotting showed that part of the recombinant protein was precipitated (Figure 5D, lane P vs. line S) and rDGAT1 was detected in the unbound fractions (Figure 5D) but undetectable in the eluted fractions (data not shown).

### SDS solubilization and purification of recombinant DGAT1 from insoluble fraction

**Figure 6:**
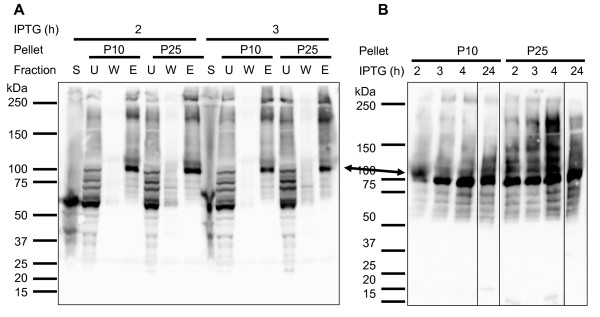
**SDS solubilization and purification of recombinant DGTA1 from insoluble fraction by Ni-NTA affinity chromatography**. Recombinant DGAT1 was induced by IPTG for various time. Cell extract was centrifuged at 10,000*g *resulting in the supernatant (S10) and the pellet (P10). S10 was further centrifuged at 25,000*g *resulted in the supernatant (S25) and the pellet (P25). P10 and P25 were solubilized by 0.4% SDS followed by centrifugation at 50,000*g *(S50). S50 and Ni-NTA purification fractions were used for detecting rDGAT1 by immunoblotting with anti-MBP-hTTP antibodies. (A) Purification of rDGAT1 from SDS-solubilized fraction. S, the supernatant S25, U, unbound, W, wash, E, elution. (B) Time course of rDGAT1 purified from SDS-solubilized fraction. The full-length rDGAT1 is marked with an arrow.

Immunoblotting showed that the great majority of rDGAT1 was recovered in the 10,000*g *pellet (Figure 4B). Therefore, SDS was used to solubilize rDGAT1 from 10,000*g *as well as the 25,000 g pellet fractions followed by purification with Ni-NTA affinity chromatography. Immunoblotting showed that full-length rDGAT1 was barely detectable in the 25,000 g supernatant and the wash (Figure 6A). Some full-length rDGAT1 and several degraded protein bands were detected in the unbound fraction and the major protein band was detected in the eluted fraction (Figure 6A). The full-length rDGAT1 was stable in cells under IPTG induction for 2-24 h as demonstrated in the purified fractions from the SDS-solubilized pellet (Figure 6B).

### Detergent and urea solubilization and purification of recombinant DGAT1 from insoluble fraction

**Figure 7:**
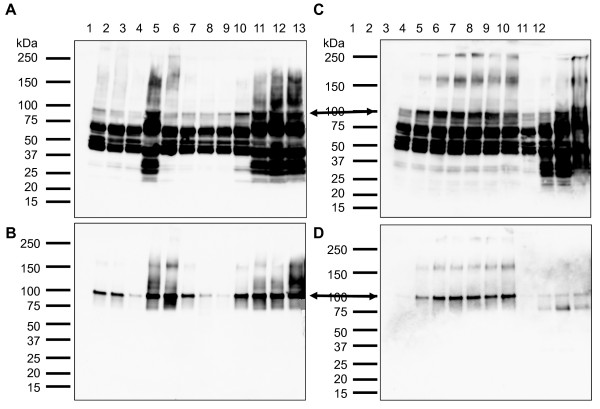
**Detergent and urea solubilization and purification of recombinant DGTA1 from insoluble fraction by Ni-NTA affinity chromatography**. The insoluble fraction (10,000*g *pellet) of *E. coli *was solubilized with 7 detergents (0.5% final concentration, 4°C, 1 h) followed by centrifugation at 20,000*g *for 10 min. The 20,000*g *supernatant was used for affinity purification with Ni-NTA beads. Recombinant DGAT1 was detected by immunoblotting using anti-MBP-hTTP antiserum. (A, C) solubilized fractions, (B, D) purified fractions. Lane 1, protein size standards; Lane 2, Brij 35; Lane 3, CHAPS; Lane 4, NP-40; Lane 5, SDS; Lane 6, Triton X-100; Lane 7, Tween 20; Lane 8, Tween 80; Lanes 9 to 13, 0, 0.1, 0.3, 0.5 and 1% SDS, respectively. (C, D) Lane 1, protein size standards, Lanes 2-8, 0, 0.1, 0.3, 0.5, 1, 1.5 and 2% Triton X-100, respectively; Lanes 9-12, 0, 2, 4 and 6 M urea, respectively. Each lane was loaded with equal amounts of proteins corresponding to those before solubilization. The full-length rDGAT1 is marked with an arrow.

As the results indicated that rDGAT1 was associated with the insoluble pellet, an attempt was made to solubilize the recombinant protein from the 10,000*g *pellet with seven different detergents (Brij 35, CHAPS, NP-40, SDS, Triton X-100, Tween 20 and Tween 80) and urea followed by purification with Ni-NTA affinity chromatography. Immunoblotting showed that SDS was the most effective detergent for rDGAT1 solubilization, while only small amounts of the full-length rDGAT1 was detected in the solubilized fractions by other detergents (Figure 7A, lanes 2-7). The optimal concentrations for SDS solubilization were 0.3-1% (Figure 7A, lanes 11-13). Ni-NTA affinity purification showed that solubilization by both SDS and Triton X-100 resulted in the highest yields of full-length rDGAT1 (Figure 7B, lanes 5-6 and 10-13). Therefore, various concentrations of Triton X-100 were tested for the solubilization of rDGAT1 from the pellet. Immunoblotting showed that 0.3-1% Triton X-100 were the optimal concentrations for the extraction (Figure 7C, lanes 2-8) and purification (Figure 7D, lanes 2-8). Urea at 4 M and 6 M also solubilized the fusion protein to a significant level (Figure 7C, lanes 9-12). However, Ni-NTA affinity chromatography indicated that rDGAT1 from urea-solubilized samples bound to the affinity beads poorly because much less of the full-length protein was obtained in the urea-solubilized protein solution (Figure 7D, lanes 9-12). Silver staining of the purified fractions indicated multiple proteins co-purified with the recombinant protein (data not shown). Extensive efforts were directed to purify rDGAT1 from the Ni-NTA purified proteins following SDS and Triton X100 solubilization. However, the protein could not be purified to near homogeneity by amylose resin affinity, Superose 12 size exclusion, and Mono Q anion exchange chromatography (data not shown).

### PreScission protease digestion of recombinant DGAT1

**Figure 8:**
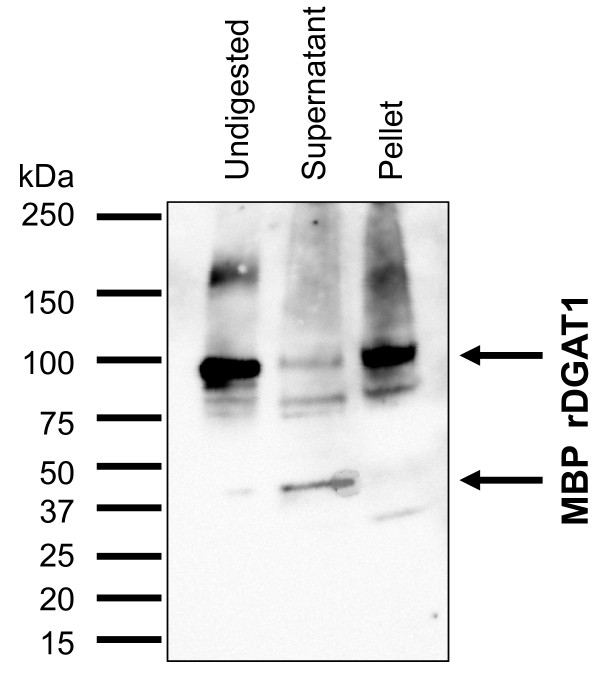
**PreScission protease digestion of recombinant DGAT1**. The Ni-NTA affinity-purified rDGAT1 was digested with PreScission protease followed by centrifugation at 10,000*g*. Equivalent amounts of protein in the undigested, the supernatant and the pellet of the digestion mixtures were separated by 4-20% SDS-PAGE and detected with anti-MBP-mTTP antibodies. The full-length rDGAT1 and the released MBP are marked with arrows.

Immunoblotting results showed that MBP-TTP antibodies detected multiple protein bands from soluble fraction (Figure 3), insoluble fraction (Figure 4B), membrane fraction (Figure 4C), Ni-NTA and amylose resin affinity-purified fractions (Figure 5), and detergent- and urea-solubilized fractions (Figures 6 and 7). To confirm the identity of the full-length rDGAT1, PreScission protease digestion was performed using rDGAT1 purified by Ni-NTA affinity chromatography (Figure 5A) because a PreScission protease cleavage site was engineered between MBP and DGAT1 fusion partners (Figure 2). The rDGAT1 sample was digested by the protease overnight followed by centrifugation at 10,000*g*. Immunoblotting showed that a protein band with the size of MBP was detected in the soluble fraction but not in the undigested protein sample (Figure 8, lane 1 vs. lane 2). It also showed that the great majority of rDGAT1 was recovered in the pellet following digestion (Figure 8, lane 2 vs. lane 3). These results confirmed the identity of rDGAT1 and showed extensive precipitation of rDGAT1 following purification.

## Discussion

Diacylglycerol acyltransferase families (DGATs) catalyze the final and rate-limiting step of triacylglycerol (TAG) biosynthesis in eukaryotic organisms. Understanding the roles of DGATs will help to create transgenic plants with value-added properties and provide information for therapeutic intervention for obesity and related diseases. Database search has identified at least 59 DGAT1 sequences from 48 organisms (Figure 1), but the expression of any DGAT1 as a full-length protein in *E. coli *had not been reported. Here a procedure was described for expressing recombinant full-length DGAT1 in a bacterial expression system. The hydrophilic amino termini of DGAT1 from mouse and oilseed rape had previously been expressed as His-tagged fusion proteins in *E. coli *[18,19]. However, the full-length DGAT1 was not expressed in *E. coli *previously because it is an integral membrane protein with 8 putative membrane-spinning domains using topology modeling [18-20], although mice DGAT1 has only three transmembrane domains using protease protections assays and indirect immunofluorescence in conjunction with selective permeabilization of cellular membranes [21]. In this study, the full-length DGAT1 was fused to MBP at the amino terminus and His-tag at the carboxyl terminus of the protein. The full-length DGAT1 was successfully expressed in *E. coli *and it is possible that the fusion to MBP contributed to the results, as MBP has been shown to increase the solubility of target proteins such as human and mouse TTP [1,4,22].

Purification of recombinant DGATs from any source represents a challenge. Only a few studies in *E. coli *have been directly related to the purification of recombinant DGATs [6]. Expression of soluble peanut DGAT (DGAT3, 42 kDa) in *E. coli *resulted in high levels of DGAT activity and the formation of labeled TAG [12]. The recombinant N-terminal region of *Brassica napus *DGAT1 (BnDGAT1_(1-116)_His_6_) was purified from *E. coli *with a predicted molecular mass of 13,278 Da, which was confirmed by MALDI-TOF mass spectrometry; however, the apparent molecular mass on SDS-PAGE was doubled and the native size was four times of the size of the monomer due to self-association. Full-length DGAT1 was not successfully expressed in *E. coli *[18,20]. In the study herein, we engineered double affinity tags for facilitating purification of recombinant DGAT1 from *E. coli*. Recombinant DGAT1 was only partially purified from the extract by either type of affinity beads or both kinds of beads, probably due to extensive precipitation (Figure 8). Preliminary attempts to measure the activity of rDGAT1 using cold oleoyl-CoA and sn-1,2-diolein did not result in visible TGA spot on TLC plates (data not shown), probably due to the low sensitivity of cold substrate. Further experiments are required to demonstrate the biological activity of rDGAT1 using radioactive oleoyl-CoA. Our current data, together with the various published reports cited here, underline the tremendous challenges that exist for the purification of recombinant full-length DGAT proteins.

Several lines of evidence support the assignment of the full-length rDGAT1 on various immunoblots despite the facts that MBP-TTP antibodies detected multiple protein bands from soluble fraction, insoluble fraction, membrane fraction, Ni-NTA and amylose resin affinity-purified fractions, and detergent- and urea-solubilized fractions. First, MBP-TTP antibodies have been well-characterized in a number of previous publications which cross-react specifically with both MBP and TTP [2,3,23-25]. Second, the detection of MBP by anti-MBP-TTP antibodies [2,3] is identical to the commercial anti-MBP antibodies [1]. Third, the size of the full-length rDGAT1 on immunoblots corresponded to the calculated size of the protein. Fourth, affinity purification with Ni-NTA beads based on the His-tag at the carboxyl terminus of rDGAT1 resulted in the full-length rDGAT1 being the most predominant band on the immunoblots (Figures 5B, 6, 7B and 7D). These results also suggest that the multiple lower molecular mass bands on immunoblots are degradation products from the carboxyl terminus of rDGAT1 because they were detected in amylose resin affinity-purified protein samples (Figure 4) but not if any in Ni-NTA affinity-purified protein samples (Figures 5B, 6, 7B and 7D). Finally, the full-length rDGAT1 was partially digested by PreScission protease resulting in the release of MBP in the soluble fraction (Figure 8).

During the purification process, we observed several lines of evidence that suggest rDGAT1 is directly or indirectly associated with other proteins, lipids, and plasma membranes. First, rDGAT1 is associated with *E. coli *plasma membranes, because the majority of DGAT1 fusion protein was detected in the 100,000*g *plasma membranes instead of the 100,000*g *cytosol (Figure 4C). Second, the partially purified proteins eluted from affinity beads contained multiple proteins even after extensive washes (Figure 5A and 5C). Finally, the purified protein samples appeared yellow in color. Gas chromatography confirmed that Ni-NTA purified rDGAT1 fraction contained fatty acids (data not shown). These observations are in agreement with several previous reports showing that DGAT is associated with lipids or lipid bodies [9,26-28], lipid droplets of cultured mouse adipocytes (equivalent to oil bodies in plant seeds) [29] and ER membranes [10]. As a positive control for the expression and purification experiments, the MBP fusion partner was massively induced by IPTG in the same type of *E. coli *and purified from the soluble fraction to near homogeneity by a single step of amylose resin affinity chromatography. Therefore, the direct or indirect association of rDGAT1 with other proteins, lipids, and plasma membranes may explain why tandem affinity beads were not effective in the purification of rDGAT1 to homogeneity.

## Conclusions

The current study reports the first procedure for expressing the full-length recombinant DGAT1 from any species using a bacterial expression system. The results suggest that recombinant DGAT1 is extensively degraded from the carboxyl terminus and is directly or indirectly associated with other proteins, lipids, and membranes. Although the protein was not purified to homogeneity in the current study, the ability to express full-length DGAT1 in *E. coli *could provide the basis for future purification of the protein.

## Methods

### Bacterial expression plasmid construction

The plasmid pMBP-DGAT1-His was engineered to express the full-length tung tree (*Vernicia fordii*) type 1 diacylglycerol acyltransferase (DGAT1, GenBank Accession No. DQ356680[10]) in *E. coli *protein expression system. The recombinant protein MBP-DGAT1-His (rDGAT1) contained MBP (maltose binding protein) at the amino terminus and 6 histidine residues (His) at the carboxyl terminus. Plasmid pMBP-hTTP, the cloning vector, was reported previously [1]. Plasmid pMBP-DGAT1-His was constructed by replacing the hTTP fragment in plasmid pMBP-hTTP with the PCR-amplified DGAT1 fragment at the *Kpn*I and *Spe*I sites (Figure 2A). A previously constructed DGAT1 plasmid was used as the template for PCR amplification of the DGAT1 DNA open reading frame [10]. The DGAT1 forward primer contained a *Kpn*I/*Asp718*I restriction enzyme recognition site followed by a PreScission protease cleavage site (5**'**-CTGTTTCAGGGTCCG-3**'**) [1] which codes for 5 amino acid residues (LFQGP) between MBP and DGAT1 protein sequences (Figure 2B). DGAT1 reverse primer contained sequence coding for a His-tag (5**'**-ATGATGATGATGATGATG-3**'**) at the carboxyl terminus of the recombinant protein (Figure 2B). The plasmid construction was confirmed by restriction enzyme digestion and DNA sequencing using the GenomeLab Dye Terminator Cycle Sequencing-Quick Start Kit and CEQ 8000 Genetic Analysis System (Beckman Coulter). The commercial plasmid pMAL-c2X (New England BioLabs) was used as an empty vector control for the expression and purification of MBP fusion partner.

### Expression of **recombinant DGAT1 **in ***E. coli***

Plasmid pMBP-DGAT1-His was transformed into the *E. coli *BL21(DE3) strain by electroporation. The optimum conditions for rDGAT1 expression were as follows: three separate single colonies were inoculated into Luria-Bertani (LB)-tetracycline (15 μg/mL) medium (LB-Tet) and grown overnight with shaking at 37°C. The overnight culture was inoculated at a 1:20 dilution into fresh medium and grown for about 4 h at 37°C to reach an optical cell density of approximately 0.6-1.0 at OD_600 nm_. Isopropylthio-β-D-galactoside (IPTG) was added to the culture medium (0.5 mM final concentration) and protein expression was induced at 25°C for up to 4 h, or in control experiments they were left uninduced for 2 h. Cells were collected by centrifugation at 5,000*g *for 10 min and homogenized by sonication in homogenization buffer (3-4 mL/g wet cells) containing amylose resin wash buffer (20 mM Tris-HCl, pH 7.4, 200 mM NaCl, 10 mM β-mercaptoethanol, 1 mM EDTA) or nickel-nitrilotriacetic agarose (Ni-NTA agarose from Qiagen) resin wash buffer (50 mM NaH_2_PO_4_, pH 7.4, 300 mM NaCl, 10 mM β-mercaptoethanol, 0.05% Tween-20), plus 0.2-1 mM phenylmethylsulfonyl fluoride (PMSF), and 1:100-1:500 dilution of protease inhibitor cocktail (Sigma, cat #P8340). The homogenate was centrifuged at 2,000*g *for 10 min to remove cell debris and the resulting supernatant was centrifuged at 10,000*g *for 10 min to remove inclusion bodies and protein aggregates [30,31]. The supernatant and the pellet were evaluated for the expression levels and solubility of rDGAT1.

### Purification of **recombinant DGAT1 **with Ni-NTA affinity chromatography

The rDGAT1 was partially purified from *E. coli *by batch method from the 10,000*g *supernatant using Ni-NTA beads according to similar procedures [2]. The 10,000*g *supernatant was mixed with Ni-NTA Agarose (Qiagen). The mixtures were incubated at 4°C with rotation for 3 h followed by centrifugation at 1,000*g *for 5 min. The beads were washed five times each with 5 bead-volume of Ni-NTA resin wash buffer. The bound proteins were eluted from the beads by gravity flow in a Bio-Rad mini-column with increasing concentrations of imidazole (Sigma) in Ni-NTA wash buffer.

### Purification of **recombinant DGAT1 with amylose resin **affinity chromatography

The rDGAT1 was subjected to purification with an amylose resin affinity column (MBPTrap HP column, GE Healthcare Life Sciences) using fast protein liquid chromatography (FPLC) (GE Healthcare Life Sciences). This system had previously been used for purification of MBP-TTP and MBP-ZFP36L1 fusion proteins [1,2,4]. The 10,000*g *supernatant or the partially purified rDGAT1 from Ni-NTA batch purification was loaded onto the column. The column was washed with 5 bed-volume of amylose resin wash buffer and then eluted with 10 bed-volume of amylose resin elution buffer (20 mM maltose in amylose resin wash buffer) followed by 0.5 M NaOH wash of the column. MBP was also affinity-purified from *E. coli *transformed with plasmid pMAL-c2x (New England Biolabs) by amylose resin affinity chromatography and used as a control in the experiments [2].

### Localization of recombinant DGAT1 in *E. coli*

*E. coli *cells were homogenized by sonication as described above. The homogenate was sequentially centrifuged at 2,000*g *for 10 min and 10,000*g *for 20 min. The 10,000*g *supernatant (2 mL) was subjected to 100,000*g *centrifugation to generate the cytosol and plasma membrane pellet [32]. The 100,000*g *pellets were suspended in 0.2 mL membrane suspension buffer containing 10 mM Tris, pH 7.4, 1 mM EDTA, and 0.2 M sucrose. Anti-MBP-hTTP antibodies were used to identify rDGAT1 in various fractions.

### Purification of recombinant DGAT1 from detergent-solubilized *E. coli *pellets

Cellular fractionation studies indicated that rDGAT1 could be recovered from the 10,000*g *and 25,000*g *pellet. Therefore, the pellets were suspended in Ni-NTA sonication buffer and used for detergent solubilization at 4°C for 1 h with 0.5% of detergents: Brij 35 (Aldrich Chemical Corp.), CHAPS (Sigma), NP-40 (Sigma), SDS (Fisher), Triton X-100 (International Biotechnologies Inc.), Tween 20 and Tween 80 (Sigma). The pellets were further solubilized at 4°C for 1 h by various concentrations of SDS (0.1, 0.3, 0.5, and 1%), Triton X-100 (0.1-2%), and urea (2-6 M). The solubilization mixtures were centrifuged at 20,000-50,000*g *for 10 min. The supernatant was used for rDGAT1 detection and purification with Ni-NTA affinity chromatography as described above.

### PreScission protease digestion of recombinant DGAT1

PreScission protease digestion was performed to confirm the identity of the full-length rDGAT1 according to a previous protocol [1]. Briefly, rDGAT1 purified by Ni-NTA affinity chromatography (Figure 5A) was mixed with PreScission protease (GE Healthcare Life Sciences). The digestion mixture was incubated at room temperature overnight with shaking followed by centrifugation at 10,000*g*. Equivalent amounts of protein in the undigested, the supernatant and pellet of the digestion mixtures were separated with 4-20% SDS-PAGE and detected with anti-MBP-mTTP antibodies.

### Protein determination, SDS-PAGE, and immunoblotting

Protein concentrations were determined with the Bradford method using the Protein Assay Dye Reagent Concentrate (Bio-Rad Laboratories) following 0.5 M NaOH treatment of the protein samples [33]. Proteins were separated by SDS-PAGE (10%, 15%, or 4-20%) and visualized by staining with Coomassie brilliant blue (Sigma) or silver staining reagent (Bio-Rad Laboratories) [2]. DGAT1 fusion protein was detected by immunoblotting following previously described procedures using nitrocellulose membranes and SuperSignal West Pico Chemiluminescent Substrate (Pierce) [2,33]. The primary antibodies were rabbit anti-MBP-hTTP antibodies [2] and anti-MBP-mTTP antibodies [3], as well as the commercial anti-MBP antibodies (New England BioLabs). The secondary antibodies were affinity-purified goat anti-rabbit IgG (H+L) horseradish peroxidase conjugate (GAR-HRP) with human IgG absorbed (Bio-Rad Laboratory).

## Lists of abbreviations

DGAT: diacylglycerol acyltransferase; His: poly histidine; IPTG: isopropylthio-β-D-galactoside; MBP: maltose binding protein; Ni-NTA: nickel-nitrilotriacetic agarose; PAGE: polyacrylamide gel electrophoresis; rDGAT1: recombinant diacylglycerol acyltransferase; TAG: triacylglycerol; TTP: tristetraprolin.

## Authors' contributions

HC conceived, designed, and performed the experiment and wrote the manuscript. DCC performed some cell culture and immunoblotting analyses. JMS and KTK are involved in providing intellectual insights and revising the manuscript. All authors read and approved the manuscript.
